# Sentinel Lymph Node Biopsy in Breast Cancer

**DOI:** 10.4021/wjon2010.01.1206

**Published:** 2010-02-01

**Authors:** Vijaya V. Mysorekar

**Affiliations:** Department of Pathology, M.S. Ramaiah Medical College, MSR Nagar, MSRIT post, Bangalore – 560 054, India. Email: vijayamysorekar@yahoo.com

**Keywords:** Breast cancer, Lymphatic mapping, Sentinel node biopsy, Micrometastases

## Abstract

The axillary lymph node status is the most reliable prognostic indicator of recurrence and overall survival in patients with breast cancer. The current standard surgical procedure for the management of invasive breast cancer is the complete removal of the cancer with total axillary clearance. However, recently, selective sentinel lymph node mapping and biopsy is gaining acceptance as a useful and accurate staging procedure, as it is minimally invasive. The sentinel lymph node is the first node into which a primary cancer drains, and is thus the first node to be involved by metastases. Patients whose sentinel nodes are negative for breast cancer metastases, can be spared a more extensive axillary lymph node dissection, with reduction in the postoperative morbidity. Sentinel node mapping is usually performed by intradermal or peritumoral injection of a combination of blue dye and radiotracer. Sentinel node examination is sometimes done intraoperatively, by imprint cytology and frozen sections, for an immediate assessment, to plan the extent of surgery at a single sitting. Permanent sections of the sentinel node are studied by serial sectioning, and immunohistochemistry for cytokeratin is done to detect micrometastases which are frequently missed on hematoxylin and eosin (H&E)-stained sections. The various aspects of sentinel node examination, and its role to decide further management in patients with ductal carcinoma-in-situ, and in other clinical settings, are discussed in this review.

## Introduction

The sentinel lymph nodes are the first nodes to receive afferent lymphatic drainage from the primary cancer. Hence, these nodes are those most likely to contain metastases. Sentinel lymph node mapping is based on the concept that if the sentinel node is negative, the other nodes of that group will also be negative in nearly all instances, whereas, if it is positive, the chance that there will be additional metastases in that nodal group is about 33% [1]. The sentinel node is now widely accepted as a reliable predictor of the axillary lymph node status in breast cancer patients. Till recently, the standard surgical procedure for all cases of invasive breast cancer was the complete removal of the cancer with total axillary clearance. However, it has now been established that axillary node dissection and its consequent morbidity can be avoided in patients in whom the sentinel nodes histologically prove to be negative for metastases. In this article, the relevant literature has been reviewed, and the clinical issues have been discussed.

## Technical aspects of lymphatic mapping

Various techniques for identifying the sentinel node, have been described. The procedure is minimally invasive, and is done under local anesthesia.

### Peritumoral injection of blue dye

Many workers use blue dye in lymphatic mapping in patients with breast cancer. This procedure, though fairly simple, requires some technical expertise. In the method described by Giuliano et al [2], 3 to 5 mL of 1% isosulphan blue is injected into the breast parenchyma around the tumor or previous biopsy site. The breast is massaged for 3-7 minutes, and the axilla is then explored until a blue-stained node along with a contiguous blue afferent lymphatic channel, or either, is identified. Blue dye travels rapidly through the lymphatics, and the sentinel node is usually highlighted within 3-10 minutes of injection [3]. However, some authors report that injection of blue dye alone is associated with decreased identification rates and a higher false-negative rate [4]. This is because of the quick transit time of the dye, on account of which it may not always remain in the node long enough for identification during surgery [5]. The other disadvantage of this technique is the inability to preoperatively identify the site of the sentinel lymph node [5].

### Peritumoral injection of radioactive colloid

This technique was first described by Krag et al [6] and subsequently by a number of workers [5, 7-9]. About 450 µCi (425 to 495 µCi) of filtered technetium-labeled (^99^m-Tc) sulfur colloid is injected at the periphery of the tumor or at the site of the previous excisional biopsy. Before making the incision, the location of greatest radioactivity is found transcutaneously using a hand-held gamma probe. The axillary incision is then made to include the area of greatest activity. The lymph nodes with the highest radioactivity counts (“hot” nodes) are removed as sentinel nodes. Although this technique is easier to perform than the blue dye method, it still needs technical expertise. Some authors feel that peritumoral injection of radioactive colloid results in significant diffusion of the radioactive tracer with the resultant background radioactivity obscuring the often slightly radioactive sentinel nodes in the axilla. This is especially problematic for upper outer quadrant tumors which form the majority of breast cancers [10].

### Peritumoral injection of both blue dye and radioactive colloid

Many studies have shown that the false-negativity rate is significantly reduced when both blue dye and radioactive colloid are used concurrently to identify the sentinel node [7, 11, 12]. With the use of a combination of blue dye and radioisotope, Cox et al [7] report a 94.4% overall success rate of detecting sentinel nodes. In this method, filtered technetium-labeled sulfur colloid is injected into the breast parenchyma 2-4 hours prior to surgery. After induction of general anesthesia, 1% isosulphan blue is injected peritumorally. A hand-held gamma probe is used to detect the location of the sentinel node prior to making the incision. The nodes which are either “hot” and “blue”, “hot” only, or “blue” only, are identified as sentinel nodes, and excised. Cody [13] compared the results of sentinel node biopsy reported by various authors using blue dye, radioisotope, or a combination of both, and found that the identification rates were 81%, 92% and 93%, respectively, while the false-negative rates were 9%, 7% and 5%, respectively. Gipponi et al [14] found that the sentinel node detection rate increased from 73.8% with blue dye alone, to 94.1% with radiotracer alone, up to 98.7% with a combination of blue dye and radiotracer. Povoski et al [15] emphasize the importance of removal of all lymph nodes showing radioactive counts or containing blue dye, in order to achieve maximum accuracy for the sentinel node biopsy procedure. In their study, a positive sentinel node was found in 18% of cases when a single sentinel node was identified, as compared to 34% when two or more sentinel nodes were identified (p = 0.003). Wong et al [16] found a false negative rate of 14.3% in patients who had only one sentinel node biopsied, as compared to 4.3% in those who had multiple sentinel nodes harvested (p = 0.0004). Thus, patients from whom only a single sentinel lymph node is harvested, are at a potential risk of being understaged and not receiving appropriate adjuvant therapy.

### Intradermal injection of blue dye and/or radioactive colloid

This method is based on the hypothesis that the mammary gland and the overlying skin share the same lymphatic drainage [17]. The procedure is simpler to perform, as compared to peritumoral injection. Intradermal injection has the advantage that lymphatic vessel density in the skin is high, and tracers are cleared more rapidly, resulting in easier detection of the sentinel nodes. Some workers have reported higher rates of successful sentinel node identification with intradermal injection [4, 18]. However, it has been stated that intradermal injection may not be suitable to detect extra-axillary nodal involvement, such as the internal thoracic (internal mammary), supraclavicular, interpectoral or intramammary nodes, to which drainage does not occur through the same lymphatic pathway as the skin [19-21]. Linehan et al [22] used intradermal radioactive colloid and intraparenchymal blue dye, and found a 95% concordance in drainage to the same node, with a 100% sentinel node identification rate.

### Subdermal injection of blue dye and/or radioactive colloid

Subdermal injection of technetium-labeled microcolloidal human serum albumin over the primary tumor, has shown a good identification rate of 97.5% [23].

### Subareolar or periareolar injection of blue dye and/or radioactive colloid

As the lymphatic flow of the breast is from superficial to deep, the lymphatic channels are in close proximity to each other in the subareolar space. Thus, injection in this region also, enables identification of the sentinel nodes. The advantage of this method is that the injection need not be image-guided, in case of non-palpable tumors. This technique has been successfully tried by various authors, with results similar to those obtained by peritumoral injection [24, 25]. Noguchi [26] recommends a combination of peritumoral injection with radioisotopes and subdermal or subareolar injection with blue dye to enhance the success rates of sentinel node identification**.**

### Preoperative breast lymphoscintigraphy

This procedure is performed about 16-24 hours prior to surgery [14, 27]. A technetium-labeled compound is injected in the skin area overlying the tumor. Planar scans of the involved breast and homolateral axilla (anterior and lateral views) are taken starting 10 minutes after injection of the radiolabeled tracer, then every 10-15 minutes up to a maximum of 2 hours. The sentinel nodes are identified and marked with the help of a skin marker. Their location may be confirmed by means of a hand-held gamma-detection probe. Preoperative lymphoscintigraphy helps to guide the surgeon for surgery, and is also useful for exploring lymph node basins other than the axilla, such as the internal thoracic (internal mammary) nodes [8, 26, 28].

Previous tissue injury or surgical disruption of the normal lymphatic drainage of the breast, may hinder successful lymphatic mapping [7]. As sentinel node mapping involves the use of radioactive materials, Cox et al [7] recommend appropriate radiation safety measures for the operative staff, surgeons, and pathologists who are participating in this investigational procedure.

## Pathological analysis

The sentinel node is excised together with a rim of surrounding tissue, and sent for histopathological examination. The pathologist has the responsibility to carefully evaluate this node for metastases. All the sentinel nodes isolated should be bisected and examined for metastases. In older individuals, fewer sentinel nodes are found, due to progressive replacement of the parenchyma of the lymph nodes by fat [28].

### Intraoperative examination

The advantage is that this method can guide surgical decision-making while sparing patients a second procedure. This can be done by touch imprint cytology which has a negative predictive value of 87-99% [10]. Another method commonly used is frozen section which allows visualization of histologic architecture. The false-negative rate of frozen section examination as compared to final histology was 13.3% in one study [14], and its negative predictive value has been reported to be between 90 to 95% [10, 14].

### Permanent histopathology sections

These are mandatory for a confirmatory assessment of the sentinel node status. The node is processed in the conventional manner. Most authors recommend step sectioning at regular intervals of about 0.25 mm to increase the chances of detecting metastases [29, 30]. Hematoxylin and eosin (H&E)-stained sections are studied for metastases.

### Immunohistochemistry for micrometastases

Micrometastases are defined as metastatic deposits between 0.2 to 2.0 mm [31]. These deposits are missed in H&E-stained sections. Immunostaining for cytokeratin is useful to detect micrometastases. The cytokeratin-positive cells can then be confirmed on H&E sections. Immunohistochemistry detects occult metastases in 12-29% of patients with T_1a_/T_1b_ tumors who are supposed to be node-negative on H&E-staining, resulting in upstaging of these patients, and, in some cases, the need for adjuvant systemic therapy [32]. Thus, undetected micrometastatic disease to the regional lymph nodes may account for a significant proportion of stage I breast cancer treatment failures.

### Reverse transcriptase-polymerase chain reaction

Reverse transcriptase-polymerase chain reaction (RT-PCR) of mRNA, only expressed in cancer cells, has the potential to detect single groups of cancer cells and can be of help in certain cases [33, 34].

Some authors suggest that a focused and detailed analysis of only the sentinel node, using the above techniques, may even improve the accuracy of axillary staging, as compared to routine histopathological processing of all lymph nodes of the axillary specimen [35, 36].

## Clinical implications

Sentinel node biopsy is a reliable and safe technique to stage the axilla. About 70 to 80% of early-stage breast cancer patients are axillary node-negative [14, 37]. When the sentinel nodes are negative for metastatic disease, the patient can be spared unnecessary dissection of all the axillary lymph nodes. The sensitivity of sentinel node biopsy for detecting metastases ranges from 83 to 100% in various studies [38]. The chances of skip metastasis, where the higher nodes in the chain are positive when the sentinel node is negative, are fortunately low [7]. In a study by Veronesi et al [39], among 167 sentinel node-negative patients who did not undergo axillary clearance, there was not a single case of overt axillary metastasis during a median follow-up period of 46 months. Similarly, in another study by Giuliano et al [40], no metastases were observed in 67 histologically sentinel node-negative breast cancer patients, over a median follow-up period of 39 months.

Patients who do not undergo axillary dissection have a better quality of life: their arm mobility is good, they do not suffer sensory numbness, and the risk of arm edema is lower when compared to those who have a complete axillary dissection [41]. Sentinel node removal may also be therapeutic because in most patients, it is the only positive axillary node [42]. A greater incidence of sentinel node positivity is found in patients with invasive tumors of larger size (more than 5 cm) [7]. The sentinel node status can influence patient management in various clinical settings as discussed below.

### Ductal carcinoma-in-situ (DCIS)

Patients with DCIS without microinvasion seldom develop regional lymph node metastasis. Despite this, recently, the use of detailed histopathologic techniques applied to sentinel lymph node examination, has resulted in the detection of sentinel node micrometastases in 12-23% of DCIS patients [43-45]. These patients are at a risk of distant metastases and may benefit by medical adjuvant treatment. However, McMasters et al [46] caution that patients with isolated tumor cells in the sentinel node should not be treated with axillary lymph node clearance, as is done in patients with macrometastases.

### Early-stage invasive mammary carcinoma

Patients with sentinel node micrometastases are at a low risk of non-sentinel node metastases, ranging from 7 to 26%. On the other hand, in patients with sentinel node macrometastases, the rate of non-sentinel node metastases is rather high, ranging from 47 to 58% [14]. Hence, in patients with early stage invasive mammary carcinoma (T_1-2_N_0_M_0_), Gipponi et al [14] propose that axillary dissection can be avoided when the sentinel node is negative or shows only micrometastases, and the primary tumor is less than or equal to 1 cm in size. They feel that axillary dissection can be reserved for patients with sentinel node micrometastases and tumor size more than 1 cm, and for those with macrometastases in the sentinel node. However, according to Noguchi [26], axillary lymph node dissection is preferable even in patients with a small tumor (T_1_) and sentinel node micrometastases, an acceptable alternative to this being radiation therapy.

### Elderly patients with small-sized tumors

Gipponi et al [14] suggest that elderly patients (above 70 years of age) who are clinically N_0_ with tumor size less than 3 cm, can be spared axillary dissection if they are sentinel node-negative. Axillary dissection can be limited to patients in the above group who are in fairly good clinical condition, with sentinel node micrometastases and tumor size more than 1 cm, or with sentinel node macrometastases. If the patient is a poor risk for general anesthesia, axillary radiation therapy can be given. Medical adjuvant therapy is also required in elderly sentinel node-positive patients.

### Prior to neoadjuvant chemotherapy in patients with large tumors

In patients with clinically N_0_ tumors larger than 3 cm, neoadjuvant chemotherapy is given to reduce the tumor load, before performing breast-conserving surgery. In such patients, sentinel node biopsy has been recommended at the time of incisional biopsy of the tumor, before starting chemotherapy. If the sentinel nodes are histologically negative for metastases, these patients could undergo a breast-saving operation alone, with post-operative axillary and chest wall radiation therapy, avoiding complete axillary dissection [14].

### Following neoadjuvant chemotherapy

Some authors recommend sentinel node biopsy as a useful procedure for staging the axilla after neoadjuvant chemotherapy in all cases, except those with inflammatory breast cancer [47-49]. However, other authors found very high false-negative rates of up to 33%, and they conclude that sentinel node biopsy is not accurate after neoadjuvant chemotherapy [10]. Hence, it has been suggested that sentinel node biopsy can be done prior to the initiation of neoadjuvant chemotherapy, and axillary dissection can be performed in sentinel node-positive patients, at the time of definitive surgery [10]. Multifocal tumors are an exclusion criterion for performing sentinel node biopsy in most centers [28].

Successful sentinel node assessment requires a multidisciplinary approach with close cooperation between nuclear medicine physicians, surgeons and pathologists [37]. False-negative biopsies may result due to surgeon inexperience or sampling error on the part of the pathologist. Hence training programs should be undertaken to train all these personnel and educate them about the importance of meticulousness in mapping, harvesting and examination of the sentinel nodes, to achieve the highest rate of accuracy.
